# Disentangling and modeling interactions in fish with burst-and-coast swimming reveal distinct alignment and attraction behaviors

**DOI:** 10.1371/journal.pcbi.1005933

**Published:** 2018-01-11

**Authors:** Daniel S. Calovi, Alexandra Litchinko, Valentin Lecheval, Ugo Lopez, Alfonso Pérez Escudero, Hugues Chaté, Clément Sire, Guy Theraulaz

**Affiliations:** 1 Centre de Recherches sur la Cognition Animale, Centre de Biologie Intégrative (CBI), Centre National de la Recherche Scientifique (CNRS) & Université de Toulouse – Paul Sabatier, 31062 Toulouse, France; 2 Groningen Institute for Evolutionary Life Sciences, University of Groningen, Centre for Life Sciences, Nijenborgh 7, 9747AG Groningen, The Netherlands; 3 Department of Physics, Massachusetts Institute of Technology, Cambridge, Massachusetts, United States of America; 4 Service de Physique de l’État Condensé, CEA – Saclay, 91191 Gif-sur-Yvette, France; 5 Computational Science Research Center, Beijing 100094, China; 6 Laboratoire de Physique Théorique, CNRS & Université de Toulouse – Paul Sabatier, 31062 Toulouse, France; Consiglio Nazionale delle Ricerche - CNR, ITALY

## Abstract

The development of tracking methods for automatically quantifying individual behavior and social interactions in animal groups has open up new perspectives for building quantitative and predictive models of collective behavior. In this work, we combine extensive data analyses with a modeling approach to measure, disentangle, and reconstruct the actual functional form of interactions involved in the coordination of swimming in Rummy-nose tetra (*Hemigrammus rhodostomus*). This species of fish performs burst-and-coast swimming behavior that consists of sudden heading changes combined with brief accelerations followed by quasi-passive, straight decelerations. We quantify the spontaneous stochastic behavior of a fish and the interactions that govern wall avoidance and the reaction to a neighboring fish, the latter by exploiting general symmetry constraints for the interactions. In contrast with previous experimental works, we find that both attraction and alignment behaviors control the reaction of fish to a neighbor. We then exploit these results to build a model of spontaneous burst-and-coast swimming and interactions of fish, with all parameters being estimated or directly measured from experiments. This model quantitatively reproduces the key features of the motion and spatial distributions observed in experiments with a single fish and with two fish. This demonstrates the power of our method that exploits large amounts of data for disentangling and fully characterizing the interactions that govern collective behaviors in animals groups.

## Author summary

We disentangle, quantify, and model the interactions involved in the control and coordination of burst-and-coast swimming in the fish *Hemigrammus rhodostomus*. We find that the interactions of fish with the arena wall result in avoidance behavior and those with a neighbor result in a combination of attraction and alignment behaviors whose effects depend on the distance and the relative position and orientation to that neighbor. Then we show that a model entirely based on experimental data confirms that the combination of these individual level interactions quantitatively reproduces the dynamics of swimming of a single fish, the coordinated motion in groups of two fish, and the consequences of interactions on their spatial distribution.

## Introduction

The study of physical or living self-propelled particles—active matter—has certainly become a booming field, notably involving biologists and physicists, often working together. Physical examples of active matter include self-propelled Janus colloids [1–8], vibrated granular matter [9–11], or self-propulsion mediated by hydrodynamical effects [12, 13], whereas biological examples are obviously ubiquitous: bacteria, cells, fish, humans, and simply speaking, most animals. In both physical and biological contexts, active matter can organize into rich collective phases. For instance, fish schools can be observed in a disordered swarming phase, or ordered schooling and vortex/milling phases [14, 15].

Yet, there are important differences between most physical and living self-propelled particles (SPP) [16]. For most physical SPP, interactions with other particles or obstacles do not modify the intrinsic or “desired” velocity of the particles but exert forces whose effect *adds up to their intrinsic velocity*. Most living SPP, like fish, birds, or humans, can also interact through physical forces (a fish or a pedestrian physically pushing another one or bumping into a wall) but mostly interact through “social forces” [17, 18]. For instance, a fish or a pedestrian wishing to avoid a physical obstacle or another individual will *modify its intrinsic velocity* in order to never actually touch it. Moreover, a physical force applied to an individual, in addition to its direct impact, can elicit a response in the form of a change in its intrinsic velocity. For instance, a fish can react to the physical force exerted by the fluid [19], resulting in a tendency for the fish to go along or against the flow. Moreover, social forces strongly break the Newtonian law of action and reaction [15, 16, 19, 20] since a fish or a pedestrian avoiding a physical obstacle obviously does not exert a contrary force on the obstacle. In addition, even between two individuals 1 and 2, the force exerted by 1 on 2 is most often not the opposite of the force exerted by 2 on 1, since social forces commonly depend on physical stimuli (light, sounds, temperature, pressure…) associated to an *anisotropic perception*: a pedestrian will most often react more to another pedestrian ahead than behind her/him. Similarly, social forces between two fish or two pedestrians also depend on their relative velocities or orientations: the need to avoid another individual will be in general greater when a collision is imminent than if it is unlikely, due to the velocity directions. Moreover, in some species of fish, individuals tend to explicitly align their heading to that of their neighbors.

Hence, if the understanding of the social interactions that underlie the collective behavior of animal groups is a central question in social ethology and behavioral ecology [21, 22], it has also a clear conceptual interest for physicists, since social and physical forces play very different roles in the dynamics of an active matter particle [16].

These social interactions play a key role in the ability of group members to coordinate their actions and collectively solve a wide range of problems, thus increasing their fitness [23–25]. In the past few years, the development of new methods based on machine learning algorithms for automating tracking and behavior analyses of animals in groups has improved to unprecedented levels the precision of available data on social interactions [26–28]. A wide variety of biological systems have been investigated using such methods, from swarms of insects [29–31] to schools of fish [32–36], flocks of birds [37–40], groups of mice [41, 42], herds of ungulates [43, 44], groups of primates [45, 46], and human crowds [47, 48], bringing new insights on behavioral interactions and their consequences on individual and collective behavior.

The fine-scale analysis of individual-level interactions opens up new perspectives to develop quantitative and predictive models of collective behavior. One major challenge is to accurately identify the contributions and combination of each interaction involved at individual-level and then to validate with a model their role in the emergent properties at the collective level [49, 50]. Several studies on fish schools have explored ways to infer individual-level interactions directly from experimental data. The force-map technique [32] and the non-parametric inference technique [33] have been used to estimate from experiments involving groups of two fish the effective turning and speeding forces experienced by an individual. In the force-map approach, the implicit assumption considers that fish are particles on which the presence of neighboring fish and physical obstacles exert “forces”. Visualizing these effective forces that capture the coarse-grained regularities of actual interactions has been a first step to characterize the local individual-level interactions [32, 33, 40]. However, none of these works incorporate or characterize the intrinsic stochasticity of individual behavior, and nor do they attempt to validate their findings by building trajectories from a model.

On the other hand, only a few models have been developed to connect a detailed quantitative description of individual-level interactions with the emergent dynamics observed at a group level [33–36]. The main difficulty to build such models comes from the entanglement of interactions between an individual and its physical and social environment. To overcome this problem, Gautrais et al. [34] have introduced an incremental approach that consists in first building from the experiments a model for the spontaneous motion of an isolated fish [51]. This model is then used as a dynamical framework to include the effects of interactions of that fish with the physical environment and with a neighboring fish. The validation of the model is then based on the agreement of its predictions with experiments on several observables in different conditions and group sizes. However, in these works, the interactions of a fish with the tank wall or another fish were only assumed to take reasonable functional forms (as a function of the fish distance and relative direction to the wall or another fish) and were depending on a few parameters which were determined by optimizing the agreement between the model predictions and the experimental results. In other words, the interactions functional forms were not truly extracted from the experimental data and a natural question which arises is whether the fair quantitative agreement of the model with experiments actually constitutes an implicit validation of the assumed forms of the interactions.

In the present work, we extend this approach to investigate the swimming behavior and interactions in the red nose fish *Hemigrammus rhodostomus*. This species performs a burst-and-coast type of swimming that makes it possible to analyze a trajectory as a series of discrete behavioral decisions in time and space. This discreteness of trajectories [36] is exploited to characterize the spontaneous motion of a fish, to identify the candidate stimuli (*e.g.* the distance, the orientation and velocity of a neighboring fish, or the distance and orientation of the tank wall), and to measure their effects on the behavioral response of a fish. We start from very general forms for the expected repulsive effect of the tank wall and for the repulsive/attractive and alignment interactions between two fish. These forms take into account the fish anisotropic perception of its physical and social environment and must satisfy some specific symmetry constraints which help us to differentiate these interactions and to disentangle and measure their relative contributions. The amount and precision of data accumulated and this modeling approach allow us to reconstruct the actual functional form of the response functions of fish governing their heading changes as a function of the distance, orientation, and angular position relative to an obstacle or a neighbor. We show that the implementation of these interactions in a stochastic model of spontaneous burst-and-coast swimming quantitatively reproduces the motion and spatial distributions observed in experiments with a single fish and with two fish. Hence, our methodology and approach are the first which combine the precise determination of the spontaneous motion and the functional forms of the interactions *and* their implementation in a synthetic and faithful model.

## Results

### Characterization of individual swimming behavior

*Hemigrammus rhodostomus* fish have been monitored swimming alone and freely in shallow water in three different circular tanks of radius *R* = 176, 250, 353 mm (see the section Materials and methods for details, and Table 1). This species performs a burst-and-coast type of swimming characterized by sequences of sudden increase in speed followed by a mostly passive gliding period (see S1 Video). This allows the analysis of a trajectory as a series of discrete decisions in time. One can then identify the candidate stimuli (*e.g.* the distance, the orientation and velocity of a neighboring fish, or the distance and orientation of an obstacle) that have elicited a fish response and reconstruct the associated stimulus-response function. Most changes in fish heading occur exactly at the onset of the acceleration phase. We label each of these increases as a “kick”.

**Table 1:** Experimental conditions.

Arena radius (mm)	1 Fish			2 Fish
	176	250	353	250
Number of experiments	11	12	8	16
Temperature (°C, mean ± se)	27.7 ± 0.2	26.8 ± 0.3	27.4 ± 0.3	26.1 ± 0.3
Body length (mm, mean ± se)	35.0 ± 1.1	28.3 ± 1.3	30.9 ± 1.5	30.3 ± 1.0

**Fig 1:**
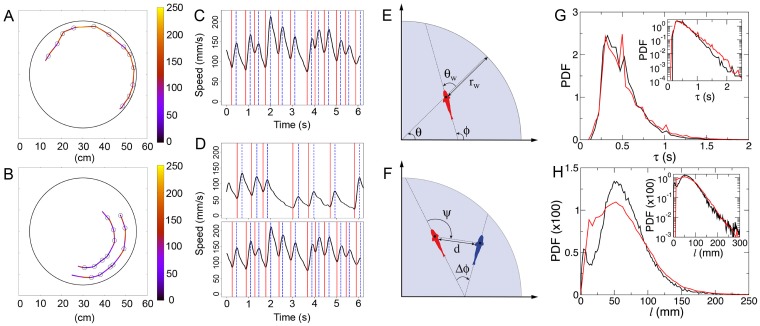
Burst-and-coast motion. Trajectories along with the bursts (circles) of a fish swimming alone (A) and a group of 2 fish (B). The color of trajectories indicates instantaneous speed. The corresponding speed time series are shown in C and D, along with the acceleration/burst phase delimited by red and blue vertical lines. E defines the variables *r*_w_ and *θ*_w_ (distance and relative orientation to the wall) in order to describe the fish interaction with the wall. F defines the relevant variables *d*, *ψ*, and Δ*ϕ* (distance, viewing angle, relative orientation of the focal fish with respect to the other fish) in order to describe the influence of the blue fish on the red one. G and H show respectively the probability distribution function (PDF) of the duration and distance traveled between two kicks as measured in the one (black) and two (red) fish experiments (tank of radius *R* = 250 mm). Insets show the corresponding graphs in semi-log scale.

Fig 1A and 1B show typical trajectories of *H. rhodostomus* swimming alone or in groups of two fish. After the data treatment (see the section Material and methods and Figs 7 and 8 there), it is possible to identify each kick (delimited by vertical lines in Fig 1C and 1D), which we use to describe fish trajectories as a group of straight lines between each of these events. While the average duration between kicks is close to 0.5 s for experiments with one or two fish (Fig 1G), the mean length covered between two successive kicks is slightly lower for two fish (Fig 1H). The typical velocity of the fish in their active periods (see Materials and methods) is of order 140 mm/s.

### Quantifying the effect of the interaction of a single fish with the wall

**Fig 2:**
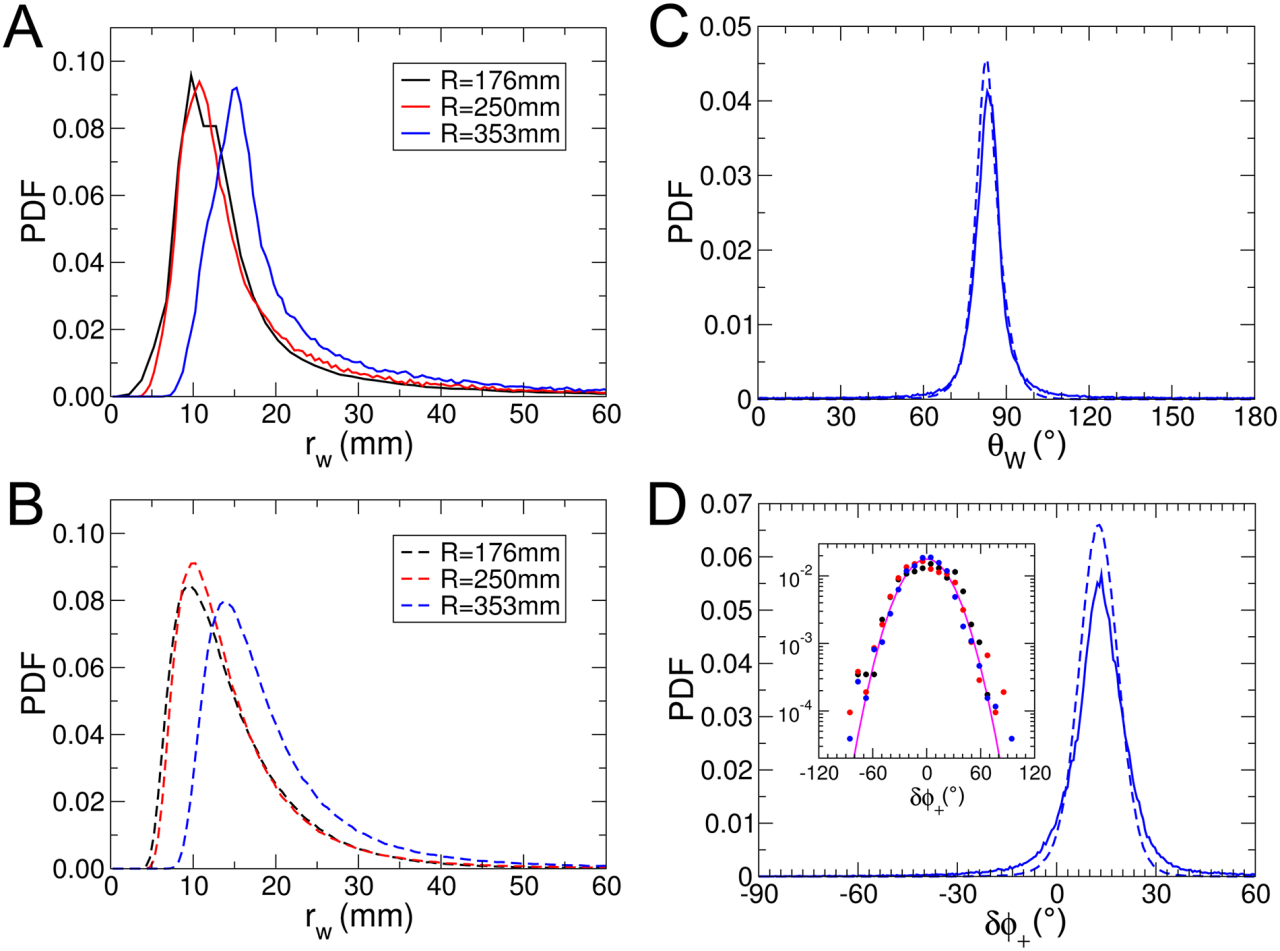
Quantification of the spatial distribution and motion of a fish swimming alone. Experimental (A; full lines) and theoretical (B; dashed lines) PDF of the distance to the wall *r*_w_ after a kick in the three arenas of radius *R* = 176, 250, 353 mm. C: experimental (full line) and theoretical (dashed line) PDF of the relative angle of the fish with the wall *θ*_w_ (*R* = 353 mm). D: PDF of the signed angle variation *δϕ*_+_ = *δϕ*×Sign(*θ*_w_) after each kick (*R* = 353 mm). The inset shows the distribution of *δϕ*_+_ when the fish is near the center of the tank (*r*_w_ > 60 mm), for *R* = 176, 250, 353 mm (colored dots), which becomes centered at *δϕ*_+_ = 0° and Gaussian of width ≈ 20° (full line).

Fig 2A shows the experimental probability density function (PDF) of the distance to the wall *r*_w_ after each kick, illustrating that the fish remains most of the time very close to the wall. We will see that the combination of the burst-and-coast nature of the trajectories (segments of average length ∼70 mm, but smaller when the fish is very close to the wall) and of the narrow distribution of angle changes between kicks (see Fig 2D) prevent a fish from efficiently escaping the curved wall of the tank (see S2 Video). Fig 2C shows the PDF of the relative angle of the fish to the wall *θ*_w_, centered near, but clearly below 90°, as the fish remains almost parallel to the wall and most often goes toward it.

In order to characterize the behavior with respect to the walls, we define the signed angle variation *δϕ*_+_ = *δϕ*×Sign(*θ*_w_) after each kick, where *δϕ* is the measured angle variation. *δϕ*_+_ is hence positive when the fish goes away from the wall and negative when the fish is heading towards it. The PDF of *δϕ*_+_ is wider than a Gaussian and is clearly centered at a positive *δϕ*_+_ ≈ 15° (tank of radius *R* = 353 mm), illustrating that the fish works at avoiding the wall (Fig 2D). When one restricts the data to instances where the fish is at a distance (*r*_w_ > 60 mm from the wall, for which its influence becomes negligible (see the section hereafter, addressing the interaction with the wall), the PDF of *δϕ*_+_ indeed becomes symmetric, independent of the tank in which the fish swims, and takes a quasi Gaussian form of width of order 20° (inset of Fig 2D). The various quantities displayed in Fig 2 will ultimately be used to calibrate and test the predictions of our model.

### Modeling and direct measurement of fish interaction with the wall

We first define a simple model for the spontaneous burst-and-coast motion of a single fish without any wall boundaries, and then introduce the fish-wall interaction, before considering the interaction between two fish in the next subsection. The large amount of data accumulated (more than 300000 recorded kicks – ∼ 100000 in each tank – for 1 fish, and 200000 for 2 fish in the *R* = 250 mm tank; see Materials and methods) permits us to not only precisely characterize the interactions, but also to provide a stringent test of the model by comparing its results to various experimental quantities (*e.g.* the full fish-wall and fish-fish distance and angle distributions instead of simply their mean, as only considered in many studies [34, 51]) which would be very sensitive to a change in the model structure and/or parameters.

#### Swimming dynamics without any interaction

**Fig 3:**
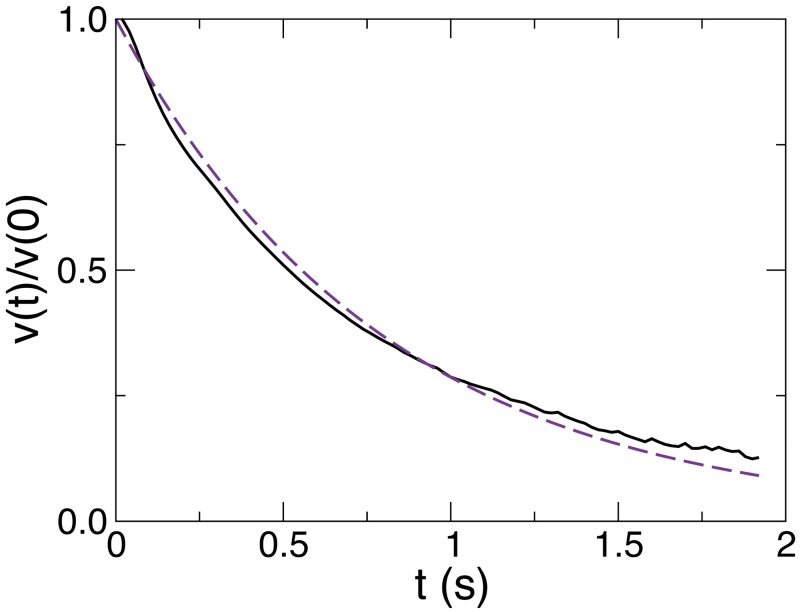
Average decay of the fish speed right after a kick. This decay can be reasonably described by an exponential decay with a relaxation time *τ*_0_ ≈ 0.80s (violet dashed line).

We model the burst-and-coast motion by a series of instantaneous kicks each followed by a gliding period where fish travel in straight lines with a decaying velocity. At the *n*-th kick, the fish located at ${\vec{x}}_{n}$ at time *t*_*n*_ with angular direction *ϕ*_*n*_ randomly selects a new heading angle *ϕ*_*n*+1_, a start or peak speed *v*_*n*_, a kick duration *τ*_*n*_, and a kick length *l*_*n*_. During the gliding phase, the speed is empirically found to decrease quasi exponentially to a good approximation, as shown in Fig 3, with a decay or dissipation time *τ*_0_ ≈ 0.80s, so that knowing *v*_*n*_ and *τ*_*n*_ or *v*_*n*_ and *l*_*n*_, the third quantity is given by $l_{n}=v_{n}\tau_{0}\left(1-\text{exp}\left[-\frac{\tau_{n}}{\tau_{0}}\right]\right)$. At the end of the kick, the position and time are updated to

$$\begin{array}{c} {\vec{x}}_{n+1}={\vec{x}}_{n}+l_{n}\vec{e}\left(\phi_{n+1}\right),\;t_{n+1}=t_{n}+\tau_{n}, \end{array}$$ (1)

where $\vec{e}\left(\phi_{n+1}\right)$ is the unit vector along the new angular direction *ϕ*_*n*+1_ of the fish. In practice, we generate *v*_*n*_ and *l*_*n*_, and hence *τ*_*n*_ from simple model bell-shaped probability density functions (PDF) consistent with the experimental ones shown in Fig 1G and 1H. In addition, the distribution of *δϕ*_R_ = *ϕ*_*n*+1_ − *ϕ*_*n*_ (the R subscript stands for “random”) is experimentally found to be very close to a Gaussian distribution when the fish is located close to the center of the tank, *i*.*e*. when the interaction with the wall is negligible (see the inset of Fig 2D). *δϕ*_R_ describes the spontaneous decisions of the fish to change its heading:

$$\begin{array}{c} \phi_{n+1}=\phi_{n}+\delta \phi_{R}=\phi_{n}+\gamma_{R}\;g, \end{array}$$ (2)

where *g* is a Gaussian random variable with zero average and unit variance, and *γ*_R_ is the intensity of the heading direction fluctuation, which is found to be of order 0.35 radian (≈ 20°) in the three tanks.

By exploiting the burst-and-coast dynamics of *H. rhodostomus*, we have defined an effective kick dynamics, of length and duration *l*_*n*_ and *τ*_*n*_. However, it can be useful to generate the full continuous time dynamics from this discrete dynamics. For instance, such a procedure is necessary to produce “real-time” movies of fish trajectories obtained from the model (see S2, S4, and S5 Videos). As already mentioned, during a kick, the speed is empirically found to decrease exponentially to a good approximation (see Fig 3), with a decay or dissipation time *τ*_0_ ≈ 0.80s. Between the time *t*_*n*_ and *t*_*n*+1_ = *t*_*n*_ + *τ*_*n*_, the viscous dynamics due to the water drag for 0 ≤ *t* ≤ *τ*_*n*_ leads to

$$\begin{array}{c} \vec{x}\left(t_{n}+t\right)={\vec{x}}_{n}+l_{n}\frac{1-\text{exp}[-\frac{t}{\tau_{0}}]}{1-\text{exp}[-\frac{\tau_{n}}{\tau_{0}}]}\vec{e}\left(\phi_{n+1}\right), \end{array}$$ (3)

so that one recovers $\vec{x}\left(t_{n}+\tau_{n}\right)=\vec{x}\left(t_{n+1}\right)={\vec{x}}_{n}+l_{n}\vec{e}\left(\phi_{n+1}\right)={\vec{x}}_{n+1}$.

#### Fish interaction with the wall

In order to include the interaction of the fish with the wall, we introduce an extra contribution *δϕ*_W_

$$\begin{array}{c} \delta \phi =\delta \phi_{R}\left(r_{w}\right)+\delta \phi_{W}\left(r_{w},\theta_{w}\right), \end{array}$$ (4)

where, due to symmetry constraints in a circular tank, *δϕ*_W_ can only depend on the distance to the wall *r*_w_, and on the angle *θ*_w_ between the fish angular direction *ϕ* and the normal to the wall (pointing from the tank center to the wall; see Fig 1E). We did not observe any statistically relevant left/right asymmetry, which imposes the symmetry condition

$$\begin{array}{c} \delta \phi_{W}\left(r_{w},-\theta_{w}\right)=-\delta \phi_{W}\left(r_{w},\theta_{w}\right). \end{array}$$ (5)

The random fluctuations of the fish direction are expected to be reduced when it stands near the wall, as the fish has less room for large angles variations (compare the main plot and the inset of Fig 2D), and we now define

$$\begin{array}{c} \delta \phi_{R}\left(r_{w}\right)=\gamma_{R}\left[1-\alpha f_{w}\left(r_{w}\right)\right]g. \end{array}$$ (6)

*f*_w_(*r*_w_) → 0, when *r*_w_ ≫ *l*_w_ (where *l*_w_ sets the range of the wall interaction), recovering the free spontaneous motion in this limit. In addition, we define *f*_w_(0) = 1 so that the fluctuations near the wall are reduced by a factor 1 − *α*, which is found experimentally to be close to 1/3, so that *α* ≈ 2/3.

If the effective “repulsive force” exerted by the wall on the fish (first considered as a physical particle) tends to make it go toward the center of the tank, it must take the form *δϕ*_W_(*r*_w_, *θ*_w_) = γ_W_ sin(*θ*_w_)*f*_w_(*r*_w_), where the term sin(*θ*_w_) is simply the projection of the normal to the wall (*i.e.* the direction of the repulsion “force” due to the wall) on the angular acceleration of the fish (of direction *ϕ* + 90°). For the sake of simplicity, *f*_w_(*r*_w_) is taken as the same function as the one introduced in Eq (6), as it satisfies the same limit behaviors. In fact, a fish does not have an isotropic perception of its environment. In order to take into account this important effect in a phenomenological way [15], we introduce *ϵ*_w_(*θ*_w_) = *ϵ*_*w*,1_ cos(*θ*_w_) + *ϵ*_*w*,2_ cos(2*θ*_w_) + …, an even function (by symmetry) of *θ*_w_, which, we assume, does not depend on *r*_w_. Finally, we define

$$\begin{array}{c} \delta \phi_{W}\left(r_{w},\theta_{w}\right)=\gamma_{W}\text{sin}\left(\theta_{w}\right)\left[1+\epsilon_{w}\left(\theta_{w}\right)\right]f_{w}\left(r_{w}\right), \end{array}$$ (7)

where *γ*_W_ is the intensity of the wall repulsion.

Once the displacement *l* and the total angle change *δϕ* have been generated as explained above, we have to eliminate the instances where the new position of the fish would be outside the tank. More precisely, and since $\vec{x}$ refers to the position of the center of mass of the fish (and not of its head) before the kick, we introduce a “comfort length” *l*_*c*_, which must be of the order of one body length (BL; 1 BL ∼ 30 mm; see Materials and methods), and we reject the move if the point $\vec{x}+\left(l+l_{c}\right)\vec{e}\left(\phi +\delta \phi \right)$ is outside the tank. When this happens, we regenerate *l* and *δϕ* (and in particular, its random contribution *δϕ*_R_), until the new fish position is inside the tank. Note that in the rare cases where such a valid couple is not found after a large number of iterations (say, 1000), we generate a new value of *δϕ*_R_ uniformly drawn in [−*π*, *π*] until a valid solution is obtained. Such a large angle is for instance necessary (and observed experimentally), when the fish happens to approach the wall almost perpendicularly to it (*δϕ* ∼ 90° or more; S5 Video at 20 s, where the red fish performs such a large angle change).

In order to measure experimentally *ϵ*_w_(*θ*_w_) and *f*_w_(*r*_w_), and confirm the functional form of Eq (7), we define a fitting procedure which is explicitly described in Materials and Methods, by minimizing the error between the experimental *δϕ* and a general product functional form *δϕ*_W_(*r*_w_, *θ*_w_) = *f*_w_(*r*_w_)*O*_w_(*θ*_w_), where the only constraint is that *O*_w_(*θ*_w_) is an *odd* function of *θ*_w_ (hence the name *O*), in order to satisfy the symmetry condition of Eq (5). Since multiplying *O*_w_ by an arbitrary constant and dividing *f*_w_ by the same constant leaves the product unchanged, we normalize *O*_w_ (and all angular functions appearing below) such that its average square is unity: $\frac{1}{2\pi }\int_{-\pi }^{+\pi }O_{w}^{2}\left(\theta_{w}\right)\;d\theta_{w}=1$.

**Fig 4:**
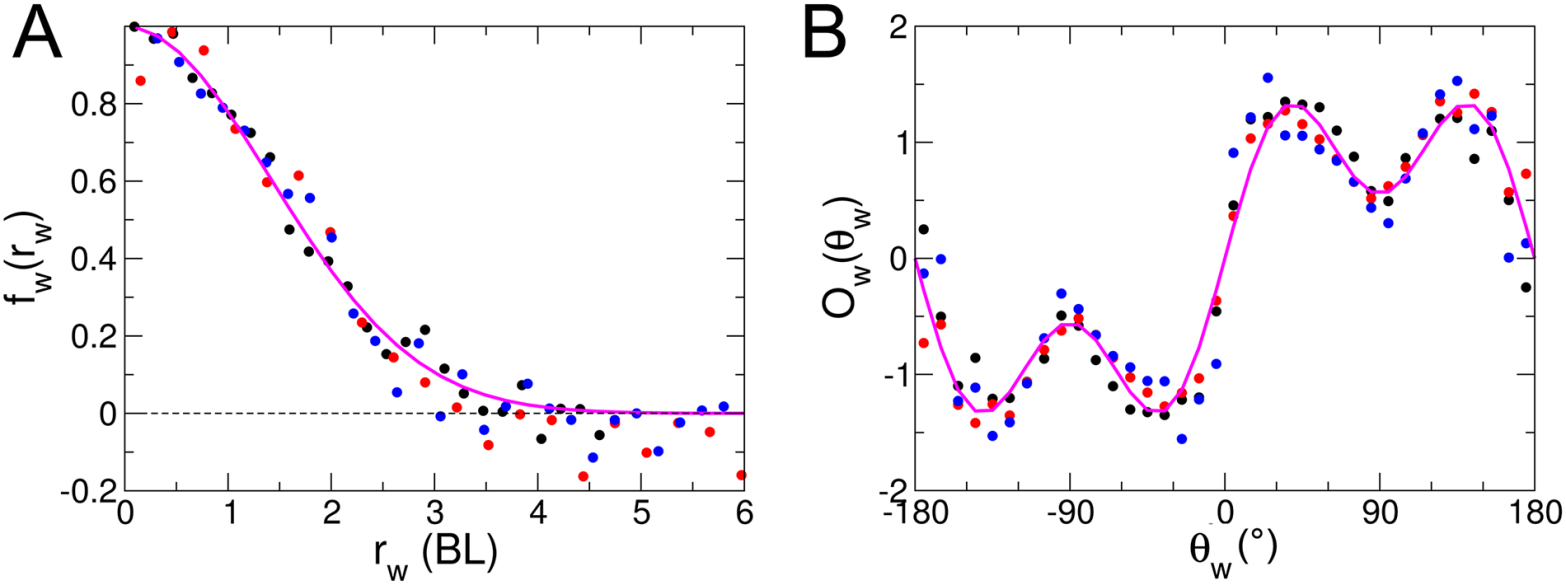
Interaction of a fish with the tank wall. Its intensity is shown as a function of the fish distance *r*_w_ (A) and relative orientation to the wall *θ*_w_ (B) as measured experimentally in the three tanks of radius *R* = 176 mm (black), *R* = 250 mm (blue), *R* = 353 mm (red). The full lines correspond to the analytic forms of *f*_w_(*r*_w_) and *O*_w_(*θ*_w_) given in the text. In particular, *f*_w_(*r*_w_) is well approximated by a Gaussian of width *l*_w_ ≈ 2 BL ∼ 60 mm.

For each of the three tanks, the result of this procedure is presented as a scatter plot in Fig 4A and 4B respectively, along with the simple following functional forms (solid lines)

$$\begin{array}{c} O_{w}\left(\theta_{w}\right)\propto \text{sin}\left(\theta_{w}\right)\left[1+0.7\text{cos}\left(2\theta_{w}\right)\right], \end{array}$$ (8)

$$f_{\text{w}}\left(r_{\text{w}}\right)=\text{exp}\left[-{\left(r_{\text{w}}/l_{\text{w}}\right)}^{2}\right],\;\text{with}\;l_{\text{w}}\approx 2\text{BL}.$$ (9)

Hence, we find that the range of the wall interaction is of order *l*_w_ ≈ 2 BL ∼ 60 mm, and is strongly reduced when the fish is parallel to the wall (corresponding to a “comfort” situation), illustrated by the deep (*i.e.* lower response) observed for *θ*_w_ ≈ 90° in Fig 4B (cos(2*θ*_w_) ≈ −1). Moreover, we do not find any significative dependence of these functional forms with the radius of the tank, although the interaction strength *γ*_*W*_ is found to decrease as the radius of the wall increases (see Table 2). The smaller the tank radius (of curvature), the more effort is needed by the fish to avoid the wall.

**Table 2:** Parameters used in the simulations.

# fish; R (mm)	*l*_*c*_ (mm)	*γ*_R_ (rad)	$\gamma_{R}^{0}$ (rad)	*γ*_W_ (rad)
1; 176	21	0.35	0.12	0.40
1; 250	30	0.35	0.14	0.12
1; 353	45	0.35	0.11	0.10
2; 250	30	0.45	0.15	0.15

Note that the fitting procedure used to produce the results of Fig 4 (described in detail in Materials and methods) does not involve any regularization scheme imposing the scatter plots to fall on actual continuous curves. The fact that they actually do describe such fairly smooth curves (as we will also find for the interaction functions between two fish) is an implicit validation of our procedure.

In Fig 2, and for the three tank radii considered, we compare the distribution of distance to the wall *r*_*w*_, relative angle to the wall *θ*_*w*_, and angle change *δϕ* after each kick, as obtained experimentally and in extensive numerical simulations of the model, finding an overall satisfactory agreement. The numerical values of the parameters of the model are listed in Table 2. On a more qualitative note, the model fish dynamics mimics fairly well the behavior and motion of a real fish (see S2 Video). The fish making only small angle changes between each kick/burst cannot escape efficiently from the curved wall, especially in the 2 smallest circular tanks.

### Quantifying the effect of interactions between two fish

Experiments with two fish were performed using the tank of radius *R* = 250 mm and a total of around 200000 kicks were recorded (see Materials and methods for details).

**Fig 5:**
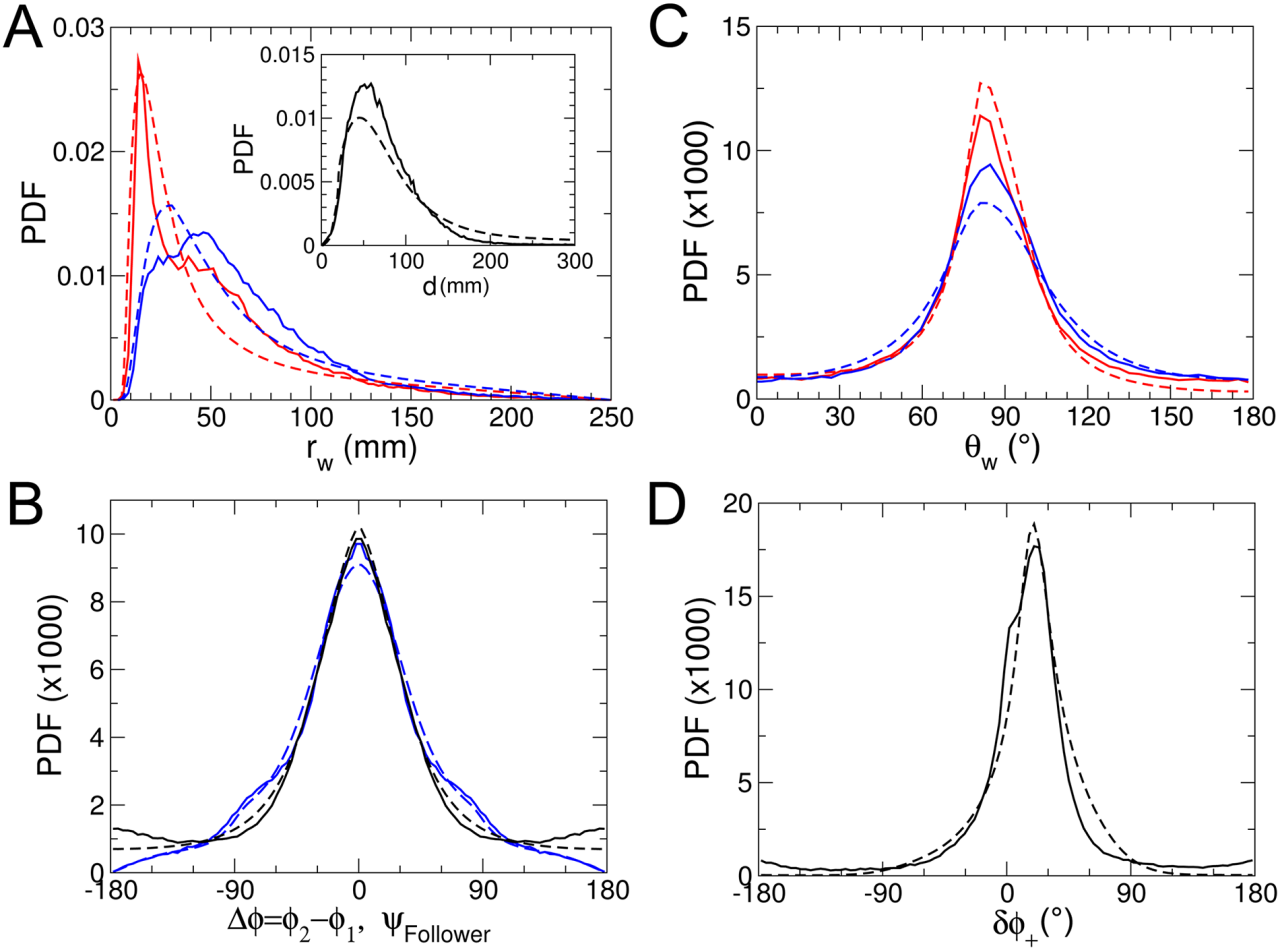
Quantification of the spatial distribution and motion in groups of two fish. In all graphs, full lines correspond to experimental results and dashed lines to numerical simulations of the model. A: PDF of the distance to the wall, for the geometrical leader (red) and follower (blue) fish; the inset displays the PDF of the distance *d* between the two fish. B: PDF of the relative orientation Δ*ϕ* = *ϕ*_2_−*ϕ*_1_ between the two fish (black) and PDF of the viewing angle *ψ* of the follower (blue). C: PDF of the relative angle to the wall *θ*_w_ for the leader (red) and follower fish (blue). D: PDF (averaged over both fish) of the signed angle variation *δϕ*_+_ = *δϕ*×Sign(*θ*_w_) after each kick.

In Fig 5, we present various experimental PDF which characterize the swimming behavior of two fish resulting from their interaction, and which will permit to calibrate and test our model. Fig 5A shows the PDF of the distance to the wall, for the geometrical “leader” and “follower” fish. The geometrical leader is defined as the fish with the largest viewing angle |*ψ*| ∈ [0, 180°] (see Fig 1F where the leader is the red fish), that is, the fish which needs to turn the most to directly face the other fish. Note that the geometrical leader is not always the same fish, as they can exchange role (see S5 Video). We find that the geometrical leader is much closer to the wall than the follower, as the follower tries to catch up and hence hugs the bend. Still, both fish are farther from the wall than an isolated fish is (see Fig 2A; also compare S2 and S4 Videos). The inset of Fig 5A shows the PDF of the distance *d* between the two fish, illustrating the strong attractive interaction between them.

Fig 5C shows the PDF of *θ*_w_ for the leader and follower fish, which are again much wider than for an isolated fish (see Fig 2C). The leader, being closer and hence more parallel to the wall, displays a sharper distribution than the follower. Fig 5B shows the PDF of the relative orientation Δ*ϕ* = *ϕ*_2_−*ϕ*_1_ between the two fish, illustrating their tendency to align, along with the PDF of the viewing angle *ψ* of the follower. Both PDF are found to be very similar and peaked at 0°. Finally, Fig 5D shows the PDF (averaged over both fish) of the signed angle variation *δϕ*_+_ = *δϕ*×Sign(*θ*_w_) after each kick, which is again much wider than for an isolated fish (Fig 2D). Due to their mutual influence, the fish swim farther from the wall than an isolated fish, and the wall constrains less their angular fluctuations.

### Modeling and direct measurement of interactions between two fish

In the presence of another fish, the total heading angle change now reads

$$\begin{array}{ccc} \delta \phi & = & \delta \phi_{R}\left(r_{w}\right)+\delta \phi_{W}\left(r_{w},\theta_{w}\right)+\\ & & \delta \phi_{\text{Att}}\left(d,\psi ,\Delta \phi \right)+\delta \phi_{\text{Ali}}\left(d,\psi ,\Delta \phi \right), \end{array}$$ (10)

where the random and wall contributions are given by Eqs (6, 7, 8, 9), and the two new contributions result from the expected attraction (“Att” subscript) and alignment (“Ali” subscript) interactions between fish. The distance between fish *d*, the relative position or viewing angle *ψ*, and the relative orientation angle Δ*ϕ* are all defined in Fig 1F. By mirror symmetry already discussed in the context of the interaction with the wall, one has the exact constraint

$$\begin{array}{c} \delta \phi_{\text{Att},\;\text{Ali}}\left(d,-\psi ,-\Delta \phi \right)=-\delta \phi_{\text{Att},\;\text{Ali}}\left(d,\psi ,\Delta \phi \right), \end{array}$$ (11)

meaning that a trajectory of the two fish observed from above the tank has the same probability of occurrence as the same trajectory as it appears when viewing it from the bottom of the tank. We hence propose the following product expressions

$$\begin{array}{c} \delta \phi_{\text{Att}}\left(d,\psi ,\Delta \phi \right)=F_{\text{Att}}\left(d\right)O_{\text{Att}}\left(\psi \right)E_{\text{Att}}\left(\Delta \phi \right), \end{array}$$ (12)

$$\begin{array}{c} \delta \phi_{\text{Ali}}\left(d,\psi ,\Delta \phi \right)=F_{\text{Ali}}\left(d\right)O_{\text{Ali}}\left(\Delta \phi \right)E_{\text{Ali}}\left(\psi \right), \end{array}$$ (13)

where the functions *O* are odd, and the functions *E* are even. For instance, *O*_Att_ must be odd as the focal fish should turn by the same angle (but of opposite sign) whether the other fish is at the same angle |*ψ*| to its left or right. Like in the case of the wall interaction, we normalize the four angular functions appearing in Eqs (12, 13) such that their average square is unity. Both attraction and alignment interactions clearly break the law of action and reaction, as briefly mentioned in the introduction. Although the heading angle difference perceived by the other fish is simply Δ*ϕ*′ = −Δ*ϕ*, its viewing angle *ψ*′ is in general not equal to −*ψ* (see Fig 1F).

As already discussed in the context of the wall interaction, an isotropic radial attraction force between the two fish independent of the relative orientation, would lead exactly to Eq (12), with *O*_Att_(*ψ*) ∼ sin(*ψ*) and *E*_Att_(Δ*ϕ*) = 1. Moreover, an alignment force tending to maximize the scalar product, *i*.*e*. the alignment, between the two fish headings takes the natural form *O*_Ali_(Δ*ϕ*) ∼ sin(Δ*ϕ*), similar to the one between two magnetic spins, for which one has *E*_Ali_(*ψ*) = 1. However, we allow here for more general forms satisfying the required parity properties, due to the fish anisotropic perception of its environment, and to the fact that its behavior may also be affected by its relative orientation with the other fish. For instance, we anticipate that *E*_Ali_(*ψ*) should be smaller when the other fish is behind the focal fish (*ψ* = 180°; bad perception of the other fish direction) than when it is ahead (*ψ* = 0°).

As for the dependence of *F*_Att_ with the distance between fish *d*, we expect *F*_Att_ to be negative (repulsive interaction) at short distance *d* ≤ *d*_0_ ∼ 1BL, and then to grow up to a typical distance *l*_Att_, before ultimately decaying above *l*_Att_. Note that if the attraction force is mostly mediated by vision at large distance, it should be proportional to the 2D solid angle produced by the other fish, which decays like 1/*d*, for large *d*. These considerations motivate us to introduce an explicit functional form satisfying all these requirements:

$$\begin{array}{c} F_{\text{Att}}\left(d\right)\propto \frac{d-d_{0}}{1+{\left(d/l_{\text{Att}}\right)}^{2}}. \end{array}$$ (14)

*F*_Ali_ should be dominant at short distance, before decaying for *d* greater than some *l*_Ali_ defining the range of the alignment interaction. For large distance *d*, the alignment interaction should be smaller than the attraction force, as it becomes more difficult for the focal fish to estimate the precise relative orientation of the other fish than to simply identify its presence.

**Fig 6:**
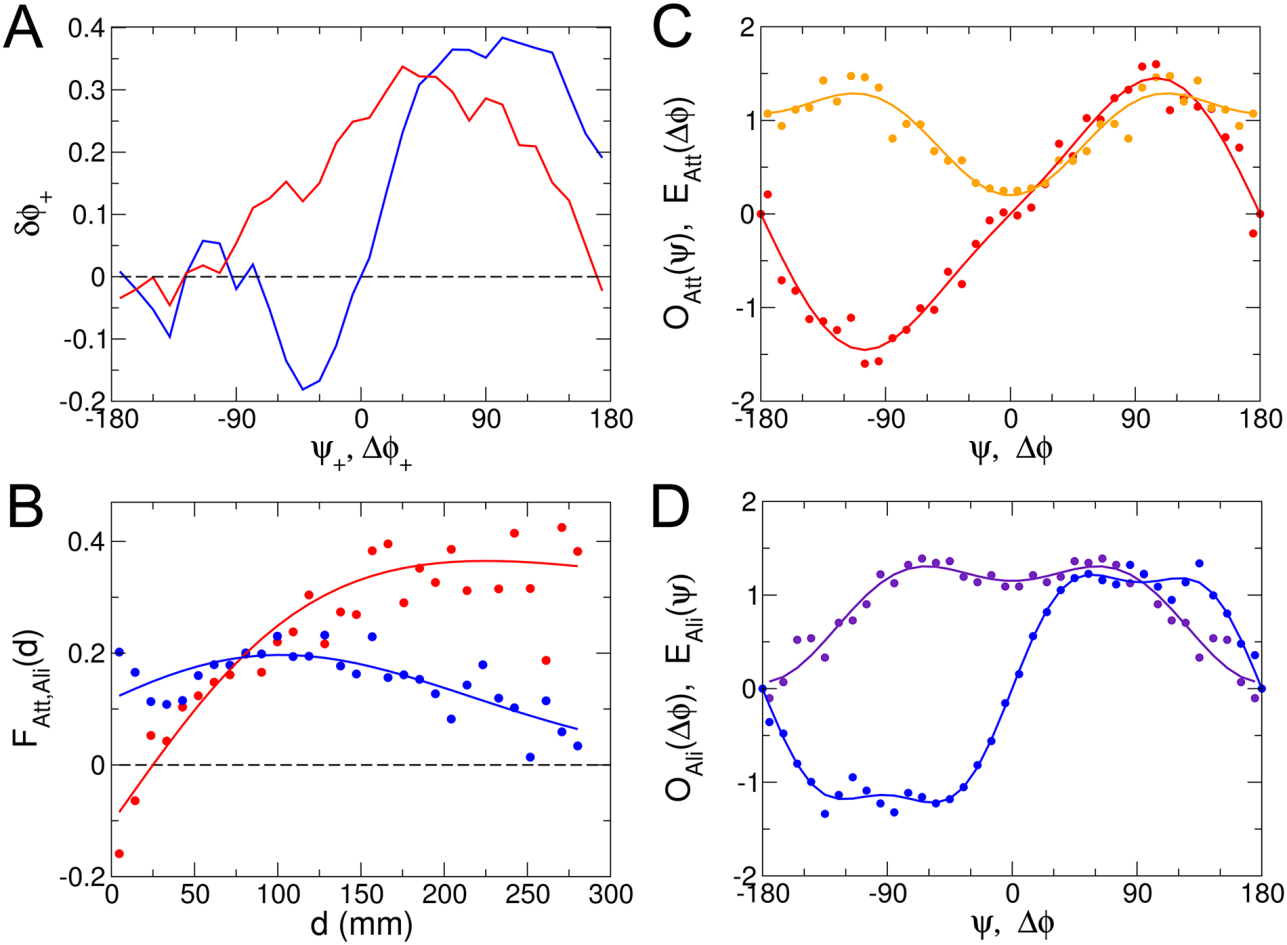
Quantification and modeling of interactions between pairs of fish. A: we plot the average signed angle change after a kick *δϕ*_+_ = *δϕ*×Sign(*ψ*) *vs* Δ*ϕ*×Sign(*ψ*) (red) and *δϕ*_+_ = *δϕ*×Sign(Δ*ϕ*) *vs* *ψ*×Sign(Δ*ϕ*) (blue) (see text). B: dependence of the attraction (*F*_Att_(*d*) in red) and alignment (*F*_Ali_(*d*) in blue) interactions with the distance *d* between fish. The full lines correspond to the physically motivated form of Eq (14) (red), and the fit proposed in the text for *F*_Ali_(*d*) (blue). C: *O*_Att_(*ψ*) (odd function in red) and *E*_Att_(Δ*ϕ*) (even function in orange) characterize the angular dependence of the attraction interaction, and are defined in Eq (12). D: *O*_Ali_(Δ*ϕ*) (odd function in blue) and *E*_Ali_(*ψ*) (even function in violet), defined in Eq (13), characterize the angular dependence of the alignment interaction. Dots in B, C, and D correspond to the results of applying the procedure explained in Materials and Methods to extract the interaction functions from experimental data.

Fig 6A shows strong evidence for the existence of an alignment interaction. Indeed, we plot the average signed angle change after a kick *δϕ*_+_ = *δϕ*×Sign(*ψ*) *vs* Δ*ϕ*×Sign(*ψ*) and δ*ϕ*_+_ = δ*ϕ*×Sign(Δ*ϕ*) *vs* *ψ*×Sign(δ*ϕ*). In accordance with Eqs (12, 13), a strong positive *δϕ*_+_ when the corresponding variable is positive indicates that the fish changes more its heading if it favors mutual alignment (reducing Δ*ϕ*), for the same viewing angle *ψ*. In other words, Fig 6A shows that the turning angle change is *amplified* when this angle change would tend to align both fish, for a given viewing angle of the focal fish and relative position of both fish.

As precisely explained in Materials and Methods, we have determined the six functions appearing in Eqs (12, 13) by minimizing the error with the measured *δϕ*, only considering kicks for which the focal fish was at a distance *r*_w_ > 2 BL from the wall, in order to eliminate its effect (see Fig 4A). This procedure leads to smooth and well behaved measured functions displayed in Fig 6. As shown in Fig 6B, the functional form of Eq (14) adequately describes *F*_Att_(*d*), with *l*_Att_ ≈ 200 mm, and with an apparent repulsive regime at very short range, with *d*_0_ ≈ 30 mm ∼ 1 BL. The crossover between a dominant alignment interaction and a dominant attraction interaction is also clear. The blue full line in Fig 6B, a guide to the eye reproducing appropriately *F*_Ali_(*d*), corresponds to the phenomenological functional form

$$\begin{array}{c} F_{\text{Ali}}\left(d\right)\propto \left(d+d_{0}^{\prime }\right)\text{exp}\left[-{\left(d/l_{\text{Ali}}\right)}^{2}\right], \end{array}$$ (15)

with *l*_Ali_ ≈ 200 mm. Note that *F*_Att_(*d*) and *F*_Ali_(*d*) cannot be properly measured for *d* > 280 mm due to the lack of statistics, the two fish remaining most of the time close to each other (see the inset of Fig 6A; the typical distance between fish is *d* ∼ 75 mm).

Fig 6C shows *O*_Att_(*ψ*) ∝ sin(*ψ*)[1 + *ϵ*_Att,1_ cos(*ψ*) + …] (odd function) and *E*_Att_(Δ*ϕ*) ∝ 1 + *η*_Att,1_ cos(Δ*ϕ*) + … (even function) along with fits involving no more than 2 non zero Fourier coefficients (and often only one; see Materials and methods for their actual values). *E*_Att_(Δ*ϕ*) has a minimum for Δ*ϕ* = 0 indicating that the attraction interaction is reduced when both fish are aligned. Similarly, Fig 6D shows *O*_Ali_(Δ*ϕ*) and *E*_Ali_(*ψ*) and the corresponding fits. As anticipated, the alignment interaction is stronger when the influencing fish is ahead of the focal fish (|*ψ*| < 90°), and almost vanishes when it is behind (*ψ* = ±180°).

In Fig 5, we compare the results of extensive numerical simulations of the model including the interactions between fish to experimental data, finding an overall qualitative (S4 and S5 Videos) and quantitative agreement.

As a conclusion of this section, we would like to discuss the generality of the product functional forms of Eqs (12, 13) for the interaction between fish, or of Eq (7) in the context of the wall interaction. As already briefly mentioned, for a physical point particle interacting through a physical force like gravity, the angle change δ*ϕ*_Att_(*d*, *ψ*) would be the projection of the radial force onto the angular acceleration (normal to the velocity of angular direction *ψ* relative to the vector between the two particles) and would then exactly take the form *F*_Att_(*d*) × sin(*ψ*). Hence, Eq (12) (resp. Eq (7), for the wall interaction) is the simplest generalization accounting for the fish anisotropic perception of its environment, while keeping a product form and still obeying the left/right symmetry condition of Eq (11) (resp. of Eq (5)). In principle, δ*ϕ*_Att_(*d*, *ψ*, Δ*ϕ*) should be written most generally as an expansion ∑_*i*_ *F*_Att,*i*_(*d*)*O*_Att,*i*_(*ψ*)*E*_Att,*i*_(Δ*ϕ*). However, as the number of terms of this expansion increases, we run the risk of overfitting the experimental data by the procedure detailed in Materials and Methods. In addition, the leading term of this expansion would still capture the main behavioral effects of the interaction and should be very similar to the results of Fig 6, while the weaker remaining terms would anyway be difficult to interpret. Note that the same argument applies to the alignment interaction, when exploiting the analogy with the magnetic alignment force between two spins. Eq (13) is the simplest generalization of the interaction *δϕ*_Ali_(*d*, Δ*ϕ*) = *F*_Ali_(*d*)sin(Δ*ϕ*) obtained in this case, while preserving the left/right symmetry and product form. Considering the fact that no regularization or smoothing procedure was used in our data analysis (see Materials and methods), the quality (low noise, especially for angular functions) of the results presented in Figs 4 and 6 strongly suggests that the generalized product forms used here capture most of the features of the actual experimental angle change.

## Discussion

Characterizing the social interactions between individuals as well as their behavioral reactions to the physical environment is a crucial step in our understanding of complex collective dynamics observed in many group-living species and their impact on individual fitness [21, 24]. In the present work, we have analyzed the behavioral responses of a fish to the presence in its neighborhood of an obstacle and to a conspecific fish. In particular, we used the discrete decisions (kicks) of *H. rhodostomus* to control its heading during burst-and-coast swimming as a proxy to measure and model individual-level interactions. The large amount of data accumulated allowed us to disentangle and quantify the effects of these interactions on fish behavior with a high level of accuracy.

We have quantified the spontaneous swimming behavior of a fish and modeled it by a kick dynamics with Gaussian distributed angle changes. We found that the interactions of fish with an obstacle and a neighboring fish result from the combination of four behavioral modes:

1. wall avoidance, whose effect starts to be effective when the fish is less than 2 BL from a wall;
2. short-range repulsion between fish, when inter-individual distance is less than 30 mm (∼1 BL);
3. attraction to the neighboring fish, which reaches a maximum value around 200 mm (∼6 to 7 BL) in our experimental conditions;
4. alignment to the neighbor, which saturates around 100 mm (∼3 BL).

In contrast to previous phenomenological models, these behavioral modes are not fixed to discrete and somewhat arbitrary zones of distances in which the neighboring fish are found [52–54]. Instead, there is a continuous combination of attraction and alignment as a function of the distance between fish. Alignment dominates attraction up to ∼75 mm (∼2.5 BL) while attraction becomes dominant for larger distances. As distance increases even more, attraction must decrease as well. However, the limited size of the experimental tanks and the lack of sufficient data for large *d* prevented us from measuring this effect, suggesting the long-range nature of the attraction interaction mediated by vision. Note that a cluster of fish can elicit a higher level of attraction, proportional to the 3D solid angle of the fish group as seen by the focal fish, as suggested by models based on visual perception [55, 56], and as captured by the power-law decay proposed in Eq (14). Designing experiments to test and quantify the long-range nature of the attraction interaction between fish would be of clear interest.

Moreover, the behavioral responses are strongly modulated by the anisotropic perception of fish. The wall repulsion effect is maximum when the orientation of the fish with regards to the wall is close to 45° and minimum when the fish is parallel to the wall. Likewise, the maximum amplitude alignment occurs when a neighboring fish is located on the front left or right and vanishes as its position around the focal fish moves towards the back.

To quantify separately the effects of attraction and alignment, we exploited physical analogies and symmetry considerations to extract the interactions between a focal fish and the wall and with another fish. Previous studies have shown that in the Golden shiners [32] and the Mosquito fish [33], there was no clear evidence for an explicit matching of body orientation. In these species, the alignment between fish was supposed to results from a combination of attraction and repulsion. However, at least in the Mosquito fish, it is likely that the strength of alignment could have been underestimated because the symmetry constraints on alignment and attraction were not taken into consideration. In the Rummy-nose tetra, we find strong evidence for the existence of an explicit alignment.

The characterization and the measurement of burst-and-coast swimming and individual interactions were then used to build and calibrate a model that quantitatively reproduces the dynamics of swimming of fish alone and in groups of two and the consequences of interactions on their spatial and angular distributions. The model shows that the wall avoidance behavior coupled with the burst-and-coast motion results in an unexpected concentration of fish trajectories close to the wall, as observed in our experiments. In fact, this phenomenon is well referenced experimentally for run-and-tumble swimming (for instance, in sperm cells [57] or bacteria [58]). It can be explained theoretically and reproduced in simple models [59, 60], as the effective discreteness of the trajectories separated in bursts or tumbles prevents the individuals from escaping the wall. Our model also reproduces the alternation of temporary leaders and followers in groups of two fish, the behavior of the temporary leader being mostly governed by its interactions with the wall, while the temporary follower is mostly influenced by the behavior of the temporary leader.

This validated model can serve as a basis for testing hypotheses on the combination of influence exerted by multiples neighbors on a focal fish in tanks of arbitrary shape. Moreover, it would certainly be interesting to study theoretically the dynamics of many fish swimming without any boundary and according to the found interactions. The study of the phase diagram as a function of the strength of the attraction and alignment interactions (and possibly their range) should show the emergence of various collective phases (schooling phase, vortex phase…) [14, 15].

Finally, our method has proved successful in disentangling and fully characterizing the interactions that govern the behavior of pairs of animals when large amounts of data are available. Hence, it could be successfully applied to collective motion phenomena occurring in various biological systems at different scales of organization.

## Materials and methods

### Experimental procedures and data collection

#### Ethics statement

Our experiments have been approved by the Ethics Committee for Animal Experimentation of the Toulouse Research Federation in Biology N°1 and comply with the European legislation for animal welfare. During the experiments, no mortality occurred.

#### Study species

**Fig 7:**
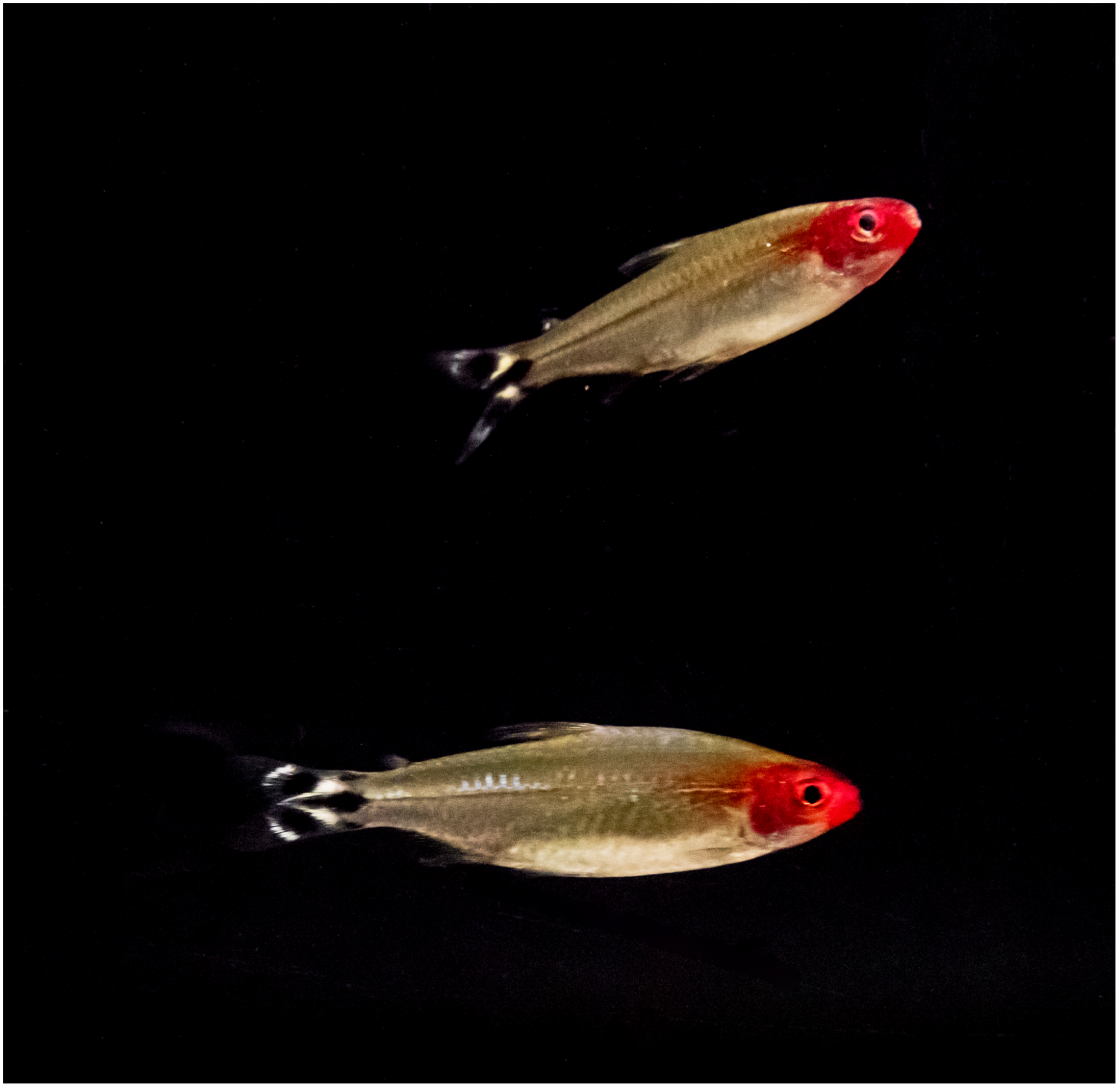
A group of two fish from the study species *Hemigrammus rhodostomus*. Credits to David Villa ScienceImage/CBI/CNRS, Toulouse, 2015.

*Hemigrammus rhodostomus* (rummy-nose tetras, Fig 7) were purchased from Amazonie Labège (http://www.amazonie.com) in Toulouse, France. This species was chosen because it exhibits a strong schooling behavior and it is very easy to handle in controlled conditions. Fish were kept in 150 L aquariums on a 12:12 hour, dark:light photoperiod, at 26.8°C (±1.6°C) and were fed *ad libitum* with fish flakes. Body lengths (BL) of the fish used in these experiments were on average 31 mm (see Table 1).

The experimental tank (120×120 cm) was made of glass and was set on top of a box to isolate fish from vibrations. The setup, placed in a chamber made by four opaque white curtains, was surrounded by four LED light panels giving an isotropic lighting. Circular tanks (of radius *R* = 176, 250, and 353 mm) were set inside the experimental tank filled with 7 cm of water of controlled quality (50% of water purified by reverse osmosis and 50% of water treated by activated carbon) heated at 26.69°C (±1.19°C) (details in Table 1). Reflections of light due to the bottom of the experimental tank are avoided thanks to a white PVC layer. Each trial started by setting one or two fish randomly sampled from their breeding tank into a circular tank. Fish were let for 10 minutes to habituate before the start of the trial. A trial consisted in one or three hours of fish freely swimming (*i.e.* without any external perturbation) in a circular tank (Table 1 and S1 Table). Fish trajectories were recorded by a Sony HandyCam HD camera filming from above the set-up at 50 Hz (50 frames per second) in HDTV resolution (1920×1080p).

Two main sources of uncertainty in the measures from video recorded from above with only one camera occur:

1. by not knowing the water depth at which a fish swims, between 0 to 7 cm from the bottom of the tank;
2. because of parallax issues (the bigger the angle between a swimming fish and the camera axis, the bigger the error made estimating the position of the fish).

The contribution of each source to the uncertainty of our measures has been estimated by computing the lengths of the cells of a chessboard set at the bottom of the tank (*Z* = 0 cm) and at the top of the water level (here *Z* = 6 cm), coming from photographs shot at two zoom levels, the one used to record experiments in the tank of radius *R* = 250 mm and the one used to record experiments in the tank of radius *R* = 353 mm. As a result, the uncertainty due to the unknown position of the fish in the water column is higher than the uncertainty due to parallax (3.5% vs 0.5%).

### Data extraction and pre-processing

Positions of fish on each frame have been tracked with the tracking software idTracker 2.1 [5]. The idTracker output format gives fish identity and barycenter positions of individuals in the image (in pixels), where the latter needs to be converted into position in the experimental frame of reference (in millimeters). The intermediary Matlab files issued by the tracker store the background image, which is the information used to calculate wall positions and thus colliding distances more accurately). Also, the intermediary files give the area of the detected fish, which can be used to determine fish heading from shape detection, independently of the trajectories. The processing of the output and intermediary files is processed with a custom-built Matlab script, which is structured into several procedures:

1. Detection of tank walls
2. Conversion to metric frame of reference
3. Fish shape detection and body length/width measurements
4. Fish activity selection and sampling
5. Segmentation
6. Segmented variables estimation

This section aims to document each of these procedures.

#### Detection of tank walls

**Fig 8:**
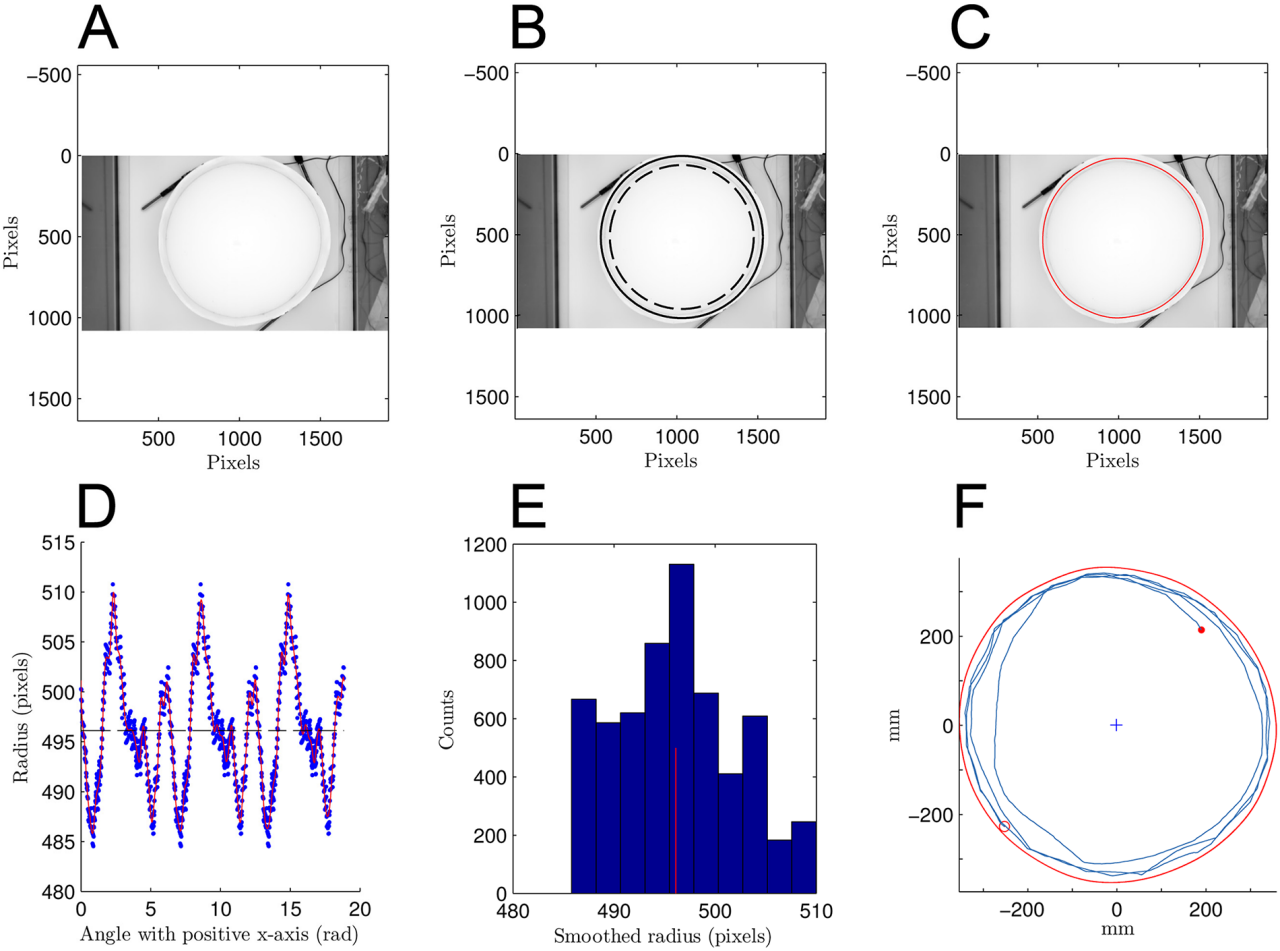
Tracking. A: Background image in grayscale extracted from a video file of an experiment with the biggest tank (radius *R* = 353 mm). B: Arena estimated from user-defined mask. The outer bold circle of radius *R* is derived from the mask drawn by the user of the tracking software and defining the area where tracking occurs. Inner dashed circle has arbitrary radius 0.85×*R*. C: Estimated walls of the tank. D: Estimation of the radius along the circle. Red line stands for cubic spline smoothing and extrapolation over 2000 angular points. The signal is repeated 3 times to improve estimation on limits (at 0 and 2*π*). The second period is kept to compute wall distances. Estimation of local polynomials is done on 30 equally spaced ranges over one period. Dashed line shows the average radius. E: Distribution of estimated radius in pixels. Red line stands for estimation of the average, used as radius approximation to compute the ratio of pixel to millimeters (PixelsToMm ratio is equal to 0.71 for this video). F: Trajectory of a fish during 40 seconds (2000 points) reported inside the estimated walls. Filled and empty circle respectively stand for start and end points.

From the tracking software idTracker, several files associated with one video are produced. In particular, a matrix is generated, with as many entries as the pixel resolution of the videos (1920×1080 pixels in our case), containing light intensity of each pixel, that is coded in a grayscale image of the video (Fig 8A). The intersection of the bottom of the tank with the tank floor is shadowed (Fig 8A). This shadow is manually enhanced to improve the detection of the tank walls.

Before running the tracking on idTracker, the user has to define a mask in order to exclude areas where individuals (here fish) cannot be tracked (e.g. outside the tank) (Fig 8B). This mask gives a raw circular estimation of the contour of the tank with radius *R* (*outer circle*) and a second raw circular estimation with arbitrary radius 0.85×*R* (*inner circle*) is derived. For *N*_*θ*_ radii with angle *θ*_*i*_ ∈ [0, 2*π*), *i* ∈ [1, 2, …, *N*_*θ*_], light intensity is measured from grayscale image every pixel from inner circle to outer circle. To measure the light intensity of a pixel, the average over the focal pixel and its 8 nearest neighbors in the image matrix is considered. We take *N*_*θ*_ = 5×360 = 1800 to oversample these measures. Mean position (*x*_*i*_, *y*_*i*_) of the three smallest values of light intensity (*i.e.* darker values) associated with each *θ*_*i*_ is computed, yielding a noisy and discrete estimate of lower image positions of the tank walls. A first smoothing procedure is run on the positions (*x*_*i*_, *y*_*i*_) to exclude bad walls estimates (e.g. detection of rust in the upper edge of the wall). A raw center (*x*_0_, *y*_0_) is defined as the mean position of all the (*x*_*i*_, *y*_*i*_) and estimate *N*_*θ*_ radii *r*_*i*_ (in pixels) for each *θ*_*i*_. The following criterion is used: two consecutive radii cannot differ by more than 5 pixels. If it is the case for the *i*^*th*^ radius, it is replaced by the previous one and associated (*x*_*i*−1_, *y*_*i*−1_) are recomputed given *θ*_*i*_ and the new radius *r*_*i*_ (in pixels). This procedure gives a new series of radii *r*_*i*_ where the previous procedure excludes outliers (Fig 8D). Assuming that fish swim at constant height *h* from the bottom, we analytically calculate from the detected positions of tank walls at the bottom the positions of the tank walls at height *h* using the formula

$$\begin{array}{c} {\vec{r}}_{h}=(1+\frac{h}{h+D})(\vec{r}-{\vec{r}}_{CCD})+{\vec{r}}_{CCD}, \end{array}$$ (16)

with *D* the metric distance between the optical center of the camera and the bottom of the tank alongside the optical axis, and *r*_*CCD*_ the position of the center of the image.

#### Conversion to metric frame of reference

A cubic spline estimation is computed to smooth the noisy signal of the radius of the detected tank (red line on Fig 8D). The radius signal is repeated over 3 periods to avoid border effects, that is to make estimations at 0 and 2*π* connected. The Matlab package immoptibox (http://www2.imm.dtu.dk/projects/immoptibox/) is used to estimate the cubic splines with function splinefit. Splines are piecewise-defined polynomial functions. Knots of the spline are chosen equally spaced. We take 30 knots on each period: the splinefit function estimates a local polynomial on each interval defined by two knots. The spline estimated over the second period (2*π* to 4*π*) is used for any subsequent calculations to ensure border continuity. The splineval function is used to find the radius of the tank wall corresponding to any angular position. Fig 8C shows positions of estimated walls for 2000 *θ* (*i.e.* of the same magnitude as the number of pixels describing the tank contours) which allow to compute the center of the tank (*x*_0_,*y*_0_) which will be used as the center of the coordinate system of fish positions. Given the mean radius derived from these estimates and the *a priori* known radius in millimeters, the number of pixels per millimeters is computed (PixelsToMm ratio) (Fig 8E). This value is the conversion ratio to translate image coordinates into the experimental metric frame of reference, which origin is taken to be the centroid of the estimated tank positions. Fig 8F exemplifies the metric fish positions and velocities. The tank coordinates are also converted to metric coordinates through the same translation and a new metric spline is evaluated to obtain the tank metric coordinates for any angular position.

#### Fish shape detection and body length/width measurement

By removing the image background information from every frame, idTracker is able to detect an approximate fish shape, *i.e.* a set of pixels’ coordinates with their corresponding light intensity, which is stored as intermediary files. These files are read to find the main axis of the fish through a principal component analysis, to estimate the typical body length (BL) and body width (BW) for every fish in all frames. BL and BW are calculated as the difference of the maximum and minimum value constituted by the projection of fish points along respectively main axis and secondary axis, then converted to metric values through the above-mentioned conversion ratio. From the main axis, a heading can be derived using the lower light intensity of fish image due to the black eye of the fish. Detection of the head direction along the main axis is done by evaluating the position relative to the barycenter of the blackest five percent of fish image points. To avoid inaccurate shape detection due to the identification of fish shade as fish shape when the fish is stopped against the wall, we approximate BL and BW as their mean value when the fish is moving faster than 15 mm.s^−1^.

#### Fish activity selection and sampling

Inter-fish variability in terms of activity is reduced by selecting the phase where a sustained swimming is observed. Considering that the observation period is much longer than the typical activity phase, these detected activity phases are sampled into sections of two minutes, allowing us to grasp the intra-fish variability occurring along an activity section. This procedure is based on the evaluation of fish speed relative to its mean body-length $u=\frac{v}{\text{BL}}$, evaluated through a centered difference scheme with 0.16 s amplitude. First, the program detects whether the fish are *swimming*, *pausing* or *stopping*. *Swimming* is defined as swimming at a speed greater than a threshold velocity *u*_min_. *Pausing* is defined as the fastest fish of the group swimming at a speed smaller than *u*_min_ for a period of time smaller or equal to *τ*_*s*_ = 4 s. *Stopping* is defined as the fastest fish of the group swimming at a speed smaller than *u*_min_ during more than *τ*_*s*_ = 4 s. The program extracts sequences of frames where the fish is either *swimming* or *pausing*, removing *stopping* behavior. From these sequences where the fish are active, *i.e.* not *stopping*, the program cuts series of the same length *τ*_*l*_. For each experiment, the program will give discontinued series where the fish are swimming. The number of series for each experiment can be used to estimate how much an experiment will participate in the statistics. The values *τ*_*l*_ = 120 s and *τ*_*s*_ = 4 s are chosen as a compromise between the amount of data available and the insensitivity of the results of activity selection to the mere parameters. The value of *u*_min_ = 0.5 BL.s^−1^ is a reasonably low threshold that allows to exclude the low activity phases where the fish uses pectoral fin swimming, of no interest for our study describing regular fish motion using body and caudal fin swimming. The proportion of time where individuals are detected active over the whole experiment is listed in S1 Table (column *Proportion of active swimming*).

#### Segmentation

*H. rhodostomus* swims in a burst-and-coast (or burst-and-glide) style. There is a succession of short acceleration phases during which the fish may also change its heading and each acceleration phase is followed by a gliding phase during which the velocity decreases and then the cycle starts again (Fig 1C, main text). The points of acceleration exhibited by fish when “bursting” is used to detect these decisions. Most heading changes occur at these decision points also called “kicks”. Our assumption is that these kicks are sufficient to describe fish swimming behavior, the passive phases containing only a consequence of the previous action, being entirely determined by physical forces. Thus, we can minimize the amount of noise given by barycenter estimation and minor trajectory deviations by describing the trajectory as segments between kicks. In order to properly identify the acceleration events, we have to smooth the raw speed time series obtained by taking the modulus of the velocity vector through a centered difference scheme over a moving time window of bandwidth 0.08 s (4 frames). We use a Savitsky-Golay [61] filter of degree three over a 0.36 s time window (18 frames) to smooth the raw time series, allowing us to classify the time series into accelerating and decelerating state [38]. To limit remaining noise, we merge any consecutive pair of accelerations separated by a deceleration lasting less than 0.08 s. We then discard any acceleration lasting less than 0.08 s as it is a too short period of time regarding the typical duration of a body motion. We assume that the times of the kicks coincide with the starting of the acceleration periods.

#### Segmented variable estimation

Assuming a fish instantaneously takes a new direction and velocity every time its body motion produces an acceleration, we reduce the full time-sampled trajectories of every two minutes activity sample *b* of a fish *Id* to a set of positions and times of interest ${\left\{{\vec{x}}_{i},t_{i}\right\}}_{b}^{Id}$ corresponding to a set of decision events. From this point of view, the statistics of interest used in both data and simulation are:

- The length and time intervals between two decision events
- The absolute and relative to rotation direction change in orientation due to a decision event
- The distance between the fish centroid and the closest point on the wall (*wall distance*)
- The top speed between decision events

and are discussed below.

Length and time intervals
  Length between two decision events is defined as the Euclidean distance between both decision points $l_{i}=∥{\vec{x}}_{i+1}-{\vec{x}}_{i}∥$. The duration *τ*_*i*_ = *t*_*i*+1_−*t*_*i*_ of the kick initiated at *t*_*i*_ is calculated from decision times.
Heading
  Heading of the fish *ϕ*_*i*_ during the kick initiated at *t*_*i*_ is identified to the direction of the vector between two decision points.
Computation of wall distances
  *θ*_*i*_, the angle in radians between the positive *x*-axis of the frame and (*x*_*i*_, *y*_*i*_) is computed from the current position of the fish (*x*_*i*_, *y*_*i*_). The radius *r*_*θ*_*i*__ for *θ*_*i*_ is computed based on the spline estimation described in the previous section. The wall distance is the Euclidean distance between fish position and estimated wall position as in $r_{w,i}=∥r_{\theta_{i}}{\vec{e}}_{\theta_{i}}-{\vec{x}}_{i}∥$.
Top speed between kicks
  The top speed *v*_*i*_ between kicks is determined from the smoothed speed time series used by the segmentation procedure, taking the maximum value reached between kicks at time *t*_*i*_ and *t*_*i*+1_.

#### Symmetrization of the data

We did not observe any statistically relevant left/right asymmetry in the distribution of angles *θ*_w_ (1 and 2 fish; see Fig 1E), or *ψ* and Δ*ϕ* (2 fish; see Fig 1F). Assuming perfect left/right symmetry amounts to saying that a trajectory as observed from the top of the tank (as we did) has exactly the same probability to occur as the very same trajectory but as seen from under the tank (“mirror trajectory”). For the mirror trajectory, all angles *θ*_w_, *ψ*, and Δ*ϕ* have the opposite sign compared to the original trajectory. Hence, the systematic angle change *δϕ* of a fish due to the interaction with the wall (1 or 2 fish experiments) and with another fish (2 fish experiments) must exactly satisfy the symmetry condition

$$\begin{array}{c} \delta \phi \left(r_{w},-\theta_{w}\right)=-\delta \phi \left(r_{w},\theta_{w}\right), \end{array}$$ (17)

for 1 fish experiments, and

$$\begin{array}{c} \delta \phi \left(r_{w},-\theta_{w},d,-\psi ,-\Delta \phi \right)=-\delta \phi \left(r_{w},\theta_{w},d,\psi ,\Delta \phi \right), \end{array}$$ (18)

for 2 fish experiments. In order to analyze and disentangle the interactions, notably the attraction and alignment interactions between fish, we have imposed general functional forms (see Eqs (7, 12, 13) in the main text) obeying these conditions. Accordingly, exploiting this assumed but reasonable left/right symmetry, we have effectively doubled our data set by adding the mirror trajectory associated to each observed trajectory. This procedure not only reduces the statistical uncertainty on quantities depending on angles (by a factor $\sqrt{2}$, by the law of large numbers), but it also helps stabilizing the optimization procedure used to extract the various components of the interactions from *δϕ*, which is detailed in the next section.

### Analysis of the interactions

#### Interaction with the wall of a single fish

The position and orientation of a fish relative to the wall is fully determined by *r*_w_ and *θ*_w_ (see the main Fig 1E). As explained below Eq (4), in addition to the random component of the angle change *δϕ* between kicks, which accounts for the fish spontaneous motion, we look for a systematic angle change due to the presence of the wall of the form *δϕ*_W_(*r*_w_, *θ*_w_) = *f*_w_(*r*_w_)*O*_w_(*θ*_w_), where *O*_w_(*θ*_w_) is an odd function of *θ*_w_. If the wall interaction tends to push back the fish toward the center of the tank and is isotropic, one has exactly that *O*_w_(*θ*_w_) ∝ sin(*θ*_w_) (the projection of a radial force on the angular acceleration, which is perpendicular to the velocity). Hence, the ansatz *δϕ*_W_(*r*_w_, *θ*_w_) = *f*_w_(*r*_w_)*O*_w_(*θ*_w_) is the simplest generalization accounting for the fish anisotropic perception of its environment, while keeping a product form and still obeying left/right symmetry. Note that in the Gautrais *et al.* model [34, 51], the sign function was phenomenologically used instead of the sin function. Despite having a qualitatively similar shape, and being both odd functions of *θ*_w_ as requested by symmetry (see above), the sign function has the unphysical/unbiological drawback of attributing a sharp discontinuous response to a fish when it approaches the wall from an arbitrary small angle from the left or the right (an angle sign that the fish could not measure with such a perfect precision).

In order to measure the actual *f*_w_(*r*_w_) and *O*_w_(*θ*_w_), we first define a discrete mesh of the two-dimensional space (*r*_w_, *θ*_w_), with each direction *r*_w_ ∈ [0, *R*] and *θ*_w_ ∈ [-*π*, *π*] partitioned respectively in *I* and *J* boxes (typical values are *I* = 40 and *J* = 30). We tabulate the unknown functions *f*_w_(*r*_w_) and *O*_w_(*θ*_w_) by defining *f*_*i*_ as the (mean) value of *f*_w_(*r*_w_) when *r*_w_ falls in box *i*, and *O*_*j*_ as the (mean) value of *O*_w_(*θ*_w_) when *θ*_w_ falls in box *j*. We finally define *ϵ*_*ij*_ as the number of data points falling in the squared box of index *i* and *j*, and *δϕ*_*ij*_ as the averaged experimental angle change for data points in this box.

*f*_*i*_ and *O*_*j*_ are then determined by minimizing the error

$$\begin{array}{c} \Delta =\sum_{i=1}^{I}\sum_{j=1}^{J}\epsilon_{ij}{\left(\delta \phi_{ij}-f_{i}O_{j}\right)}^{2}. \end{array}$$ (19)

This minimization is achieved by writing the equation ∂Δ/∂*f*_*i*_ = 0 and ∂Δ/∂*O*_*j*_ = 0 under the form

$$\begin{array}{c} f_{i}=\frac{\sum_{j=1}^{J}\epsilon_{ij}\delta \phi_{ij}O_{j}}{\sum_{j=1}^{J}\epsilon_{ij}O_{j}^{2}}, \end{array}$$ (20)

$$\begin{array}{c} O_{j}=\frac{\sum_{i=1}^{I}\epsilon_{ij}\delta \phi_{ij}f_{i}}{\sum_{i=1}^{I}\epsilon_{ij}f_{i}^{2}}. \end{array}$$ (21)

It is straightforward to realize that if the experimental *δϕ*_*ij*_ were exactly of the form *f*_*i*_×*O*_*j*_, the right-hand side of Eqs (20, 21) would indeed exactly recover *f*_*i*_ and *O*_*j*_.

This system is solved iteratively starting from reasonable initial conditions, but most importantly with *O*_*j*_ being an odd function of *θ*_w_. We checked that this procedure leading to the results of Fig 4 does not depend on the initial conditions.

In practice, knowing the *f*_*i*_’s and *O*_*j*_’s at a given iteration, we generate their values at the next iteration by computing

$$\begin{array}{ccc} f_{i}^{\prime } & = & \left(1-p\right)f_{i}+p{\hat{f}}_{i}, \end{array}$$ (22)

$$\begin{array}{ccc} O_{j}^{\prime } & = & \left(1-p\right)O_{j}+p{\hat{O}}_{j}, \end{array}$$ (23)

where ${\hat{f}}_{i}$ and ${\hat{O}}_{j}$ are given by the right-hand side of Eqs (20, 21), and *p* is a damping parameter that we took equal to 0.25. Obviously, the fixed point solution of Eqs (22, 23) ultimately coincides with the wanted solution of Eqs (20, 21).

Since multiplying *O*_*j*_ by an arbitrary constant and dividing *f*_*i*_ by the same constant leaves the product *f*_*i*_ *O*_*j*_ unchanged, we choose to normalize *O*_*j*_ (and all angular functions appearing in Figs 4 and 6) after each iteration such that its average square is unity:

$$\begin{array}{c} \frac{1}{J}\sum_{j=1}^{J}O_{j}^{2}=\frac{1}{2\pi }\int_{-\pi }^{+\pi }O_{w}^{2}\left(\theta_{w}\right)\;d\theta_{w}=1 \end{array}$$ (24)

The procedure described here converges to a relative accuracy of 10^−6^ in typically 100 iterations, leading to the result of Fig 4.

#### Attraction and alignment interaction between two fish

We define a procedure identical in spirit as above, but involving more unknown interaction functions now depending on the 3 parameters *d*, *ψ*, and Δ*ϕ* defined in Fig 1F. We choose to restrict our analysis to data points for which the focal fish was at a distance greater than 2 BL∼60 mm, for which the wall interaction is found to be negligible by the previous analysis.

Again, we partition the three-dimensional space (*d*, *ψ*, Δ*ϕ*) in a mesh of *K*×*L*×*M* boxes (with typically *K* = 40, *L* = *M* = 30). As previously, we define *ϵ*_*klm*_ as the number of data points falling in the cubic box of index *k*, *l* and *m*, and *δϕ*_*klm*_ as the averaged experimental angle change of the focal fish for data points in this box.

As explained below Eq (11), the systematic angle change *δϕ* = *δϕ*_Att_(*d*, *ψ*, Δ*ϕ*) + *δϕ*_Ali_(*d*, *ψ*, Δ*ϕ*) due to the attraction and alignment forces is parameterized by 6 unknown functions

$$\begin{array}{c} \delta \phi_{\text{Att}}\left(d,\psi ,\Delta \phi \right)=F_{\text{Att}}\left(d\right)O_{\text{Att}}\left(\psi \right)E_{\text{Att}}\left(\Delta \phi \right), \end{array}$$ (25)

$$\begin{array}{c} \delta \phi_{\text{Ali}}\left(d,\psi ,\Delta \phi \right)=F_{\text{Ali}}\left(d\right)O_{\text{Ali}}\left(\Delta \phi \right)E_{\text{Ali}}\left(\psi \right), \end{array}$$ (26)

with parity constraints (*O* functions are odd, *E* functions are even). These rather general functional forms translate into mathematical forms the notion of attraction and alignment which can in fact have cumulative or contrary effects depending on the relative position or orientation of the two fish (see Fig 6A). Intuitively, attraction means that if the other fish is on the right, the focal fish should turn to the right, and should turn by the same amount to the left in the “mirror situation” (see the “Symmetrization of the data” section above in Materials and methods).

Again we tabulate these 6 functions, depending each on only one variable, as *F*_Att,*k*_, *O*_Att,*l*_, *E*_Att,*m*_, *F*_Ali,*k*_, *O*_Ali,*m*_, *E*_Ali,*l*_. These functions are determined by minimizing the error

$$\begin{array}{ccc} \Delta = & & \sum_{k=1}^{K}\sum_{l=1}^{L}\sum_{m=1}^{M}\epsilon_{klm}\times (\delta \phi_{klm}-F_{\text{Att},k}O_{\text{Att},l}E_{\text{Att},m}\\ & & -F_{\text{Ali},k}O_{\text{Ali},m}E_{\text{Ali},l}{)}^{2}. \end{array}$$ (27)

Expressing that the derivative of Δ with respect to the 6 tabulated function is zero at the minimum, we obtain 6 equations similar to Eqs (20, 21), although a bit more complicated. For instance, *F*_Att,*k*_ satisfies the fixed point equation

$$\begin{array}{c} F_{\text{Att},k}=\frac{\sum_{l,m}\epsilon_{klm}O_{\text{Att},l}E_{\text{Att},m}\left(\delta \phi_{klm}-F_{\text{Ali},k}O_{\text{Ali},m}E_{\text{Ali},l}\right)}{\sum_{l,m}\epsilon_{klm}O_{\text{Att},l}^{2}E_{\text{Att},m}^{2}}. \end{array}$$ (28)

Note the presence of the counter term *F*_Ali,*k*_ *O*_Ali,*m*_ *E*_Ali,*l*_ between the parentheses of Eq (28), meaning that the attraction force is evaluated by subtracting the estimated alignment interaction to the actual experimental angle change. Again, it is straightforward to check that if the experimental *δϕ*_*klm*_ takes exactly the form *F*_Att,*k*_ *O*_Att,*l*_ *E*_Att,*m*_ + *F*_Ali,*k*_ *O*_Ali,*m*_ *E*_Ali,*l*_, Eq (28) exactly recovers the correct *d* dependence of the attractive force *F*_Att,*k*_.

Finally, the resulting system of 6 equations is solved by the same iterative procedure as for the wall interaction (including the normalization of the average square of angular functions), leading to the results of Fig 6B, 6C, and 6D. In particular, the angular functions obtained are plotted with simple analytical forms given by

$$\begin{array}{ccc} O_{\text{Att}}\left(\psi \right) & \propto & \text{sin}(\psi )[1-0.33\text{cos}(\psi )], \end{array}$$ (29)

$$\begin{array}{ccc} E_{\text{Att}}\left(\Delta \phi \right) & \propto & 1+0.48\text{cos}(\Delta \phi )-0.31\text{cos}(2\Delta \phi ), \end{array}$$ (30)

$$\begin{array}{ccc} O_{\text{Ali}}\left(\Delta \phi \right) & \propto & \text{sin}(\Delta \phi )[1+0.30\text{cos}(2\Delta \phi )], \end{array}$$ (31)

$$\begin{array}{ccc} E_{\text{Ali}}\left(\psi \right) & \propto & 1+0.60\text{cos}(\psi )-0.32\text{cos}(2\psi ), \end{array}$$ (32)

the overall multiplicative constant being fixed by the normalization of the average square of these functions to unity. The analytical forms for *F*_Att_(*d*) and *F*_Ali_(*d*) are respectively discussed above Eqs (14) and (15).

One of the main interests of assuming these reasonable product forms is to drastically limit the number of fitting parameters, but also to derive from these forms an *explicit* model. Indeed, if we were to produce a three-dimensional map of *δϕ* on the *K*×*L*×*M* mesh made of 36000 boxes (*K* = 40, *L* = *M* = 30), we would need 36000 fitting parameters at this resolution, barely smaller than the number of kicks available from experiment (∼ 200000 for 2 fish). The present procedure only requires 2×(*K* + *L* + *M*) = 200 fitting parameters, which allows us to extract the main feature of the interactions with a high resolution and yet a rather small noise (see Figs 4 and 6, and in particular, the various angular functions).

### Parameter estimation and simulations

As shown in the inset of Fig 2D, the spontaneous angle change has a nearly Gaussian distribution of zero mean (left/right symmetry) and variance *γ*_R_ ≈ 0.35 rad, as measured when the fish is far from the wall (*r*_w_ > 2 BL). This value of *γ*_R_ was used in all simulations of the one fish dynamics, in the three circular tanks of radius *R* = 176, 250, 353 mm. For two fish, *γ*_R_ is found experimentally to be slightly larger, probably because each fish activity is stimulated by the presence of the other fish.

The total angle change PDF shown in the main graph of Fig 2D (one fish) and in Fig 5D (two fish) is dominated by the majority of kicks where the fish are very close to the wall, and has a width reduced by a factor nearly 3, so that a fair estimate of the spontaneous angle change intensity near the wall is (see Eq (6))

$$\begin{array}{c} \gamma_{R}^{0}=\left(1-\alpha \right)\gamma_{R}∼\gamma_{R}/3. \end{array}$$ (33)

The exact value of $\gamma_{R}^{0}$ (or *α*), of the comfort distance to the wall *l*_*c*_ introduced below Eq (7), which should naturally be of order of 1 BL ∼30 mm, and of the intensity of the interaction with the wall *γ*_W_ are tuned near their experimental expected value, resulting in the very satisfactory agreement with experiments obtained in Figs 2 and 5. The values of the parameters used in our simulations are reported in Table 2.

For each of the 4 conditions (the three tank sizes for one fish and one tank size with two fish; see S1 Table), the graphs of Fig 2 (one fish) and in Fig 5 (two fish) are obtained by typically simulating 100 runs with 10^6^ kicks each, compared to the 100000-200000 kicks recorded experimentally for each condition/tank.

## Supporting information

S1 Table List of experiments. (PDF)

S1 Video One fish trajectories. Left: A typical experiment with 1 fish swimming in a circular tank of radius 250 mm. Right: 3D representation of the tracking and analysis output. The successive positions of the fish are represented by spheres whose color depends on its current speed (maximum and minimum speeds are shown in red and light blue respectively). The xy plane represents the position of fish as seen in the top down recording, and the z axis corresponds to the time evolution in the last 5 s. Newer positions appear at the top, while positions older than the aforementioned threshold are erased from the animation.
(MOV)

S2 Video Experiment vs model—one fish. Comparison of the dynamics of a single fish in an experiment (left) and in a typical run of the model (right; starting from approximately the same location as in the experiment), in a circular tank of radius 250 mm. The animated fish size is not strictly proportional to its actual dimensions, but used to facilitate visualization. The kick-segmented dynamics obtained in the model was exploited to produce a continuous trajectory by the procedure detailed in the main text.
(MOV)

S3 Video Two fish trajectories. Left: A typical experiment with 2 fish swimming in a circular tank of radius 250 mm. Right: 3D representation of the tracking and analysis output. The successive positions of fish are represented by spheres whose color depends on their current speed (maximum and minimum speeds are shown in red and light blue respectively). The xy plane represents the position of fish as seen in the top down recording, and the z axis corresponds to the time evolution in the last 5 s. Newer positions appear at the top, while positions older than the aforementioned threshold are erased from the animation.
(MOV)

S4 Video Experiment vs model—two fish. Comparison of the dynamics of groups of two fish in an experiment (left) and in a typical run of the model (right), in a circular tank of radius 250 mm (see also the legend of S1 Video for details). On the short 40 s time scale of both videos, fish display a greater variety of behaviors than for one fish experiments/simulations: wide excursions and crossing of the tank, change from clockwise to anticlockwise motion along the wall. Both videos also illustrate the fact that the geometrical leader (the red fish; see main text) can persist for a long time, although leader changes are observed experimentally and in the model on longer time scales (see S5 Video).
(MOV)

S5 Video Leader change. This video illustrates an exchange (at 20 s) of geometrical leader and follower in a typical run of the model with two fish in a circular tank of radius 250 mm. Note that the large angle change of the red fish at 20 s is probably due to the rejection procedure detailed in the main text, sometimes leading to such a large spontaneous random angle change *δϕ*_*R*_ when a fish arrives almost perpendicularly to the wall.
(MOV)
