# Supplementary material for: Disentangling and modeling interactions in fish with burst-and-coast swimming reveal distinct alignment and attraction behaviors
Source: PLoS Comput Biol. 2018 Jan 11;14(1):e1005933. doi: 10.1371/journal.pcbi.1005933 (PMC5783427; doi:10.1371/journal.pcbi.1005933)
Supplement: S1 Table — (PDF) [file pcbi.1005933.s001.pdf]

# Supporting information

**S1 Table. List of experiments.**

| Number of fish | Tank Size (mm) | Date | Proportion of active swimming | Duration (min) | Number of kicks |
|---|---|---|---|---|---|
| 1 | 176 | 2014-08-20 | 0.8 | 191 | 21012 |
| | | 2014-08-20 | 0.1 | 193 | 2572 |
| | | 2014-08-21 | 0.4 | 187 | 10122 |
| | | 2014-08-21 | 0.7 | 197 | 19849 |
| | | 2014-08-22 | 0.7 | 204 | 15691 |
| | | 2014-08-22 | 0.1 | 202 | 2401 |
| | | 2014-08-26 | 0.9 | 230 | 31603 |
| | | 2014-08-26 | 0.1 | 208 | 1550 |
| | | 2015-07-18 | 0.0 | 188 | 631 |
| | | 2015-07-21 | 0.2 | 178 | 4459 |
| | | 2015-07-25 | 0.5 | 185 | 11772 |
| | 250 | 2014-04-25 | 0.1 | 186 | 3199 |
| | | 2014-04-25 | 0.3 | 191 | 6112 |
| | | 2014-04-29 | 0.3 | 195 | 6363 |
| | | 2014-04-29 | 0.2 | 222 | 5955 |
| | | 2014-04-30 | 0.5 | 187 | 13632 |
| | | 2014-05-06 | 0.3 | 181 | 6760 |
| | | 2014-05-06 | 0.1 | 191 | 2206 |
| | | 2014-05-07 | 0.5 | 187 | 12127 |
| | | 2015-06-10 | 0.6 | 190 | 10584 |
| | | 2015-07-19 | 0.5 | 185 | 11531 |
| | | 2015-07-28 | 0.2 | 204 | 3833 |
| | | 2015-07-31 | 0.3 | 198 | 7179 |
| | 353 | 2014-05-12 | 0.5 | 205 | 13180 |
| | | 2014-05-13 | 0.7 | 207 | 16325 |
| | | 2014-05-13 | 0.4 | 192 | 11362 |
| | | 2014-05-28 | 0.2 | 197 | 4468 |
| | | 2015-07-23 | 0.2 | 187 | 6501 |
| | | 2015-07-26 | 0.3 | 189 | 6357 |
| | | 2015-07-29 | 0.8 | 202 | 21944 |
| | | 2015-07-30 | 0.9 | 209 | 23604 |
| 2 | 250 | 2013-01-31 | 0.9 | 65 | 13605 |
| | | 2013-02-04 | 0.9 | 65 | 14473 |
| | | 2013-02-13 | 1.0 | 41 | 9037 |
| | | 2013-03-04 | 0.9 | 68 | 13663 |
| | | 2013-03-14 | 0.0 | 86 | 1151 |
| | | 2013-03-18 | 0.9 | 61 | 12401 |
| | | 2013-03-27 | 0.9 | 63 | 14331 |
| | | 2014-02-14 | 1.0 | 62 | 13365 |
| | | 2014-03-11 | 1.0 | 61 | 15012 |
| | | 2014-11-04 | 0.9 | 80 | 15287 |
| | | 2014-11-05 | 0.3 | 77 | 6136 |
| | | 2014-11-05 | 0.7 | 79 | 11910 |
| | | 2014-11-05 | 0.5 | 75 | 8458 |
| | | 2014-11-06 | 0.5 | 75 | 9262 |
| | | 2014-11-06 | 0.8 | 78 | 14732 |
| | | 2014-11-07 | 0.7 | 81 | 12904 |
